# Linking young men who have sex with men (YMSM) to STI physicians: a nationwide cross-sectional survey in China

**DOI:** 10.1186/s12879-018-3145-2

**Published:** 2018-05-18

**Authors:** Bolin Cao, Peipei Zhao, Cedric Bien, Stephen Pan, Weiming Tang, Julia Watson, Guodong Mi, Yi Ding, Zhenzhou Luo, Joseph D. Tucker

**Affiliations:** 1 School of Media and Communication, Shenzhen University, Shenzhen, China; 2 University of North Carolina Project – China, Guangzhou, China; 3 Shenzhen Nanshan Chronic Disease Control Center, Shenzhen, China; 4 School of Medicine, University of North Carolina at Chapel Hill, Chapel Hill, North Carolina USA; 5 Blued, Beijing, China; 6 London School of Hygiene and Tropical Medicine, London, UK

**Keywords:** MSM, Social media, HIV, STIs, China

## Abstract

**Background:**

Many young men who have sex with men (YMSM) are reluctant to seek health services and trust local physicians. Online information seeking may encourage YMSM to identify and see trustworthy physicians, obtain sexual health services, and obtain testing for sexually transmitted infections (STIs). This study examined online STI information seeking behaviors among Chinese YMSM and its association with offline physician visits.

**Methods:**

We conducted a nationwide online survey among YMSM through WeChat, the largest social media platform in China. We collected information on individual demographics, sexual behaviors, online STI information seeking, offline STI testing, and STI physician visits. We examined the most commonly used platforms (search engines, governmental websites, counseling websites, generic social media, gay mobile apps, and mobile medical apps) and their trustworthiness. We assessed interest and willingness to use an MSM-friendly physician finder function embedded within a gay mobile app. Logistic regression models were used to examine the correlation between online STI information searching and offline physician visits.

**Results:**

A total of 503 men completed the survey. Most men (425/503, 84.5%) searched for STI information online. The most commonly used platform to obtain STI information were search engines (402/425, 94.5%), followed by gay mobile apps (201/425, 47.3%). Men reported high trustworthiness of information received from gay mobile apps. Men also reported high interest (465/503, 92.4%) and willingness (463/503, 92.0%) to use a MSM-friendly physician finder function within such apps. Both using general social media (aOR =1.14, 95%CI: 1.04–1.26) and mobile medical apps (aOR =1.16, 95%CI: 1.01–1.34) for online information seeking were associated with visiting a physician.

**Conclusion:**

Online STI information seeking is common and correlated with visiting a physician among YMSM. Cultivating partnerships with the emerging mobile medical apps may be useful for disseminating STI information and providing better physician services to YMSM.

## Background

Young men who have sex with men (YMSM) are disproportionately affected by HIV and other sexually transmitted infections (STIs), yet are often reluctant to obtain healthcare services [1–4]. YMSM, between the ages of 16 and 30, may be at higher risk of HIV acquisition than older MSM because they may be more likely to be sexually active, suffer from power imbalances in relationships, and report higher rates of condomless sex and substance use [5]. As a result, YMSM require more evidence-based, MSM-friendly healthcare services. Decentralization of healthcare services may improve service quality and coverage [6]. Decentralization refers to transferring clinical services away from a single organizational authority and towards multiple sites. However, service decentralization may also result in inconsistent quality of health services [7], making it difficult to meet the unique needs of YMSM. Physician mistrust and fear of discrimination also discourage YMSM from obtaining health services [3, 4], and thus may affect timely treatment and linkage to care [2]. In order to improve sexual health services and reduce HIV/STI transmission, linkage to care of YMSM to MSM-friendly physicians is urgently needed.

YMSM report inadequate HIV/STI information and insufficient healthcare seeking [8]. The proportion of MSM who visit a STI physician in clinic is low; only 36% of MSM with suspected STI have sought STI treatment in clinics in China [9]. YMSM attending an STI clinic may experience dual stigma stemming from same-sex practices and the STI treatment-seeking [10]. Missed office visits are also common for MSM living with HIV, leading to a higher likelihood of poor HIV-related outcomes [11]. At the same time, YMSM are increasingly using the Internet to seek health information [12, 13], particularly HIV/STI information [14, 15]. This may be an opportunity for enhancing the delivery of HIV/STI information. Online information can empower YMSM with STI knowledge and encourage them to visit a physician [16–18]. Some studies have found that individuals who seek sexual health information online show an increase in safe sex behavior [17, 19], HIV/STI testing [20, 21], and service access [22].

When seeking health information online, multiple platforms are available. Search engines are a commonly used platform to initiate the searching process [23, 24]. Governmental websites offer official and authoritative health information [25, 26]. Counseling websites allow users to ask or answer health-related questions. In contrast, social media platforms allow users to generate health information available for searching. Social media platforms have also grown to include gay mobile apps and mobile medical apps, which characterize two-way interaction and emphasize connection [27]. Among online informational platforms, social media is emerging as an effective method of disseminating health information [28, 29]. Social media is an online platform that facilitates the creation and sharing of information via virtual communities and networks, and features participation, openness, conversation, and connectedness [27]. Social media can be used to collect the wisdom of crowds, build peer-mentored communities, and deliver HIV/STI prevention services [30]. Social media use is particularly prevalent among adolescents and young adults; and in some contexts, up to 30 % of young people use social media to obtain health information [13]. Social media-based health interventions targeting MSM have been found to be feasible and acceptable among YMSM at high risk [31, 32].

China has a large population of YMSM who use online information seeking platforms. Over 695 million people in China use social media [33], and gay-specific social media platforms such as “Blued” have over 27 million users in China [21]. Chinese YMSM have an HIV and syphilis prevalence of 6.7 and 8.3%, respectively [34]. This is likely related to low HIV/STD knowledge [35], high rates of unprotected anal intercourse [36], perceived low risk of HIV infection, and fears of being stigmatized [37]. Chinese YMSM are increasingly using online resources to seek partners as well as obtain sexual health information [38]. The aim of this study is to examine the relationship between online STI information seeking and offline STI physician visits among YMSM. We also sought to examine the role of social media as a source of STI information and as a method of linking care to STI healthcare providers.

## Methods

### Study design and participants

We conducted a cross-sectional, nationwide online survey from May 27 to May 30, 2017. A convenience sample of participants were recruited through three subscription accounts on the social media platform WeChat, the most popular platform in China. The three accounts included the official account of Blued, the largest gay app with 28 million active users, and the official accounts of two large HIV prevention organizations in China (Qingtong in North China and SESH in South China). Participants accessed the survey through a link attached to a HIV-related article on each WeChat account.

We developed the survey following the Checklist for Reporting Results of Internet E-Surveys (CHERRIES) [39]. The survey questionnaire was field-tested by twenty YMSM in April 2017, and their feedback was incorporated in finalizing the survey questionnaire. Field test data were not included in the final dataset for analysis.

Eligible men were born biologically male, between 16 and 30 years old, had anal or oral sex with men, and had seen a physician in the last 24 months. Physician visits were defined as seeing a physician in clinic for any reason. We chose to recruit participants with physician visiting experiences in the past 24 months so they were able to provide and recall their physician visiting behaviors. Participants were asked to sign an electronic informed consent form when starting the survey, and participants received a small phone credit reimbursement (~$7.50 USD) as a reward upon the completion of survey.

### Measures

Demographic characteristics measured in the study included age, level of education (high school or below, some college, college and above), annual income, current residence (urban or rural), occupation (student or non-student), and sexual orientation (gay, bisexual, heterosexual, or other). Participants were asked about their sexual behaviors, including the number of sex partners in the last three months and condomless anal intercourse.

We examined STI testing history and STI physician visiting behavior as outcomes in this study. STI testing history was measured by whether participants had ever tested for HIV, syphilis, hepatitis B, or hepatitis C (Yes/ No). Having tested for at least one of four sexually transmitted diseases was considered as having STI testing history (Yes). STI physician visiting behavior was measured by whether participants had ever visited a physician for any STI or HIV (Yes/No).

Participants were asked whether they had searched for STI information online in the past 24 months (Yes/NO). If yes, they were asked details regarding the online platforms used, searching on symptoms or searching for services, and perceived trustworthiness of the searching results. Online platforms for STI information were categorized into 6 groups: search engines (i.e., Google, Baidu), governmental websites (i.e., Center for Disease Control websites), counseling websites (i.e., 39 HealthNet), generic social media (i.e., WeChat, Weibo), gay mobile apps (i.e. Blued, Grindr) and mobile medical apps (i.e. Pingan Good Doctor App). The survey also included two questions regarding willingness and interest in use of a MSM-friendly doctors finding function in gay mobile Apps. MSM-friendly physicians referred to physicians who meet essential competencies for serving MSM, focusing on prevention and treatment in the survey [40].

### Statistical analysis

We conducted descriptive analyses of participant characteristics and online information searching behaviors. We used bivariate and multivariate logistic regression to examine the association between online health information seeking and offline physician visits. Multivariate logistical analyses adjusted variables including age, education, and income.

## Results

### Participant characteristics

In total, 26,952 individuals viewed the HIV-related article on WeChat, 1689 men clicked the online survey link, and 503 eligible respondents completed the survey. Of the 503 men, the average age was 23.9 ± 3.5 years old. In our study, 20.3% of men were between ages 16 and 20, 44.3% between 21 and 25, and 35.4% between 26 and 30, compared with 33.9, 35.8, and 30.2%, respectively, in the 2010 national census [41]. Geographically, participants in our study were from, in a descending order of frequency: Eastern China (61.6%, *n* = 310), Western China (17.9%, *n* = 90), Central China (14.3%, *n* = 72), Northeast China (6.2%, n = 31), compared with 40.2, 25.8, 26.8, and 7.2% respectively, in the national census [42]. Most of them lived in urban areas (85.9%), had a college degree or above (45.3%), had an annual income less than CNY 60,000 (USD 9200, 79.5%), and self-identified as gay (83.5%).

Around three-quarters (73.0%) of participants had no more than one sex partner in the past 3 months and 75.3% used condom in the last sex encounter. Most (91.5%) participants had tested for at least one type of STI in their lifetime, 85.7% had tested for HIV at least once, and 55.1% had tested for syphilis at least once, 64.2% had ever tested for Hepatitis B and 41.0% had ever tested for Hepatitis C. Of the participants, 57.9% visited a STI physician in the past 24 months (Table 1).

**Table 1:** Demographical and behavioral characteristics of YMSM participants who had visited a physician in the past 24 months in China, 2017 (*n* = 503)

Characteristics	Total	
	n	%
Age (Mean: 23.92 and SD:3.54)		
16–20	102	20.3
21–25	223	44.3
26–30	178	35.4
Education		
High school/below	134	26.6
Some college	141	28.0
College/bachelors above	228	45.3
Annual income (USD)		
< 2700	111	22.1
2700–5500	116	23.1
5501–9200	173	34.4
9201–15,000	65	12.9
> 15,000	38	7.6
Residency		
Rural	71	14.1
Urban	432	85.9
Occupation		
Student	173	34.4
Non-student	330	65.6
Sexual orientation		
Gay	420	83.5
Bisexual	60	11.9
Heterosexual	3	0.6
Unsure/other	20	4.0
Number of anal sex partners in the past 3 months		
0–1	367	73.0
Multiple	136	27.0
Used a condom during last MSM sexual encounter^a^		
Yes	357	75.3
No	117	24.7
Ever HIV tested		
Yes	431	85.7
No	72	14.3
HIV serostatus		
Positive	73	14.5
Negative	350	69.6
Never tested or Not got results	80	15.9
Ever syphilis tested		
Yes No	277 226	55.1 44.9
Syphilis serostatus		
Positive	40	8.0
Negative	237	47.1
Never tested	226	44.9
Ever Hepatitis B tested		
Yes	323	64.2
No	180	35.8
Ever Hepatitis C tested		
Yes	206	41.0
No	297	59.0
Online STI information seeking		
Yes	425	84.5
No	78	15.5
Interest in using the MSM-friendly physician function in a gay mobile App		
Very Interested	313	62.2
Somewhat interested	152	30.2
Somewhat not interested	36	7.2
Very not interested	2	0.4
Willingness to use the MSM-friendly physician function in a gay mobile App		
Yes	463	92.0
No	40	8.0
Ever STI tested ^b^		
Yes	460	91.5
No	43	8.5
Visited a STI physician in the past 24 months^c^		
Yes	291	57.9
No	212	42.1
Searching platforms^d^		
Search engine	402	94.5
Governmental websites	156	36.7
Counseling websites	72	16.9
Generic social media	151	35.5
Gay mobile app	201	47.3
Mobile medical app	50	11.8

^a^The total number for condom use was 474, as 29 participants did not have anal sex with other men.

^b^STI testing included HIV, syphilis, hepatitis B &C.

^c^STI physician visits included visiting a physician for STI and HIV.

^d^The total number of YMSM who searched STI information online was 425, which was the denominator of this item

### Interest and willingness of using a MSM-friendly physician finding function

Almost all men (92.4%) indicated they were at least somewhat interested in using a MSM-friendly physician finding function. Only 8% (38/503) participants were not interested. 92.0% (463/503) were willing to use this kind of function within gay networking apps.

### Online information seeking behavior

Among participants who had completed the survey, 84.5% (425/503) had searched for STI information online. A majority of the participants (94.5%) had used search engines, roughly half (47.3%) had used gay mobile apps, and 35.5% had used generic social media. Roughly one in ten men (11.8%) had used mobile medical apps to search for STI information.

Among those who sought STI information online, perceived trustworthiness of sexual health information from different platforms was highest for governmental websites (85.3%), and gay mobile apps (77.1%). Most participants used generic social media platforms (94.0%) and gay mobile apps (90.5%) to seek information for symptoms. In addition, nearly two thirds used gay mobile apps (65.2%) to search for sexual health services (Table 2).

**Table 2:** Approaches for YMSM participants to seek for health information online in China, 2017 (*n* = 425)

Platforms	Perceived Trustworthiness	Seeking about symptoms^a^	Seeking for services^b^
	N(%)	N(%)	N(%)
Search engine	243/402(60.4%)		
Governmental websites	133/156(85.3%)		
Counseling websites	54/72(75.0%)		
Generic social media	93/151(61.6%)	142/151 (94.0%)	115/151 (76.2%)
Gay mobile App	155/201(77.1%)	182/201 (90.5%)	131/201 (65.2%)
Mobile medical App^c^	37/50(74.0%)	44/50(88.0%)	40/50(80.0%)

^a^Symptoms included latest search about STIs and symptoms of STIs;

^b^Services included viewing online comments on physicians and making an appointment.

^c^Mobile medical apps referred to apps that provide medical counselling and treatment service, such as Ping An Good Doctor App and Good Doctor Online App

### Factors associated with offline physician visiting behavior

Binary logistic regression results showed that generic social media use (OR = 1.18, 95% CI: 1.08–1.29) and gay mobile (OR = 1.10, 95% CI: 1.01–1.19), instead of mobile medical app use (OR = 1.11, 95%CI = 0.97–1.26), were significantly associated with STI physician visits. In multivariate logistic analyses, generic social media use (aOR =1.14, 95% CI: 1.04–1.26) and mobile medical app use (aOR =1.16, 95%CI: 1.01–1.34) were significantly associated with STI physician visits (Table 3). In addition, the binary logistic regression results showed online seeking for symptoms (aOR =2.50, 95%CI: 1.74–3.60) and services (aOR =2.42, 95%CI: 1.65–3.54) were correlated with offline STI physician visits. The multivariate analyses also suggested that both online seeking for symptoms (aOR =2.02, 95%CI: 1.34–3.04) and services (aOR =1.95, 95%CI: 1.28–2.99) were correlated with STI physician visit offline.

**Table 3:** Multivariate logistical regression on health information seeking on social media with offline physician-visiting behaviors of YMSM participants in China, 2017 (*n* = 503)

	STI testing^a^	STI physician visiting^b^		
	OR 95% CI	aOR^c^ 95% CI	OR 95% CI	aOR 95% CI
Platform use				
Generic social media use	1.44** (1.10–1.88)	1.39* (1.06–1.81)	1.18*** (1.08–1.29)	1.14** (1.04–1.26)
Gay mobile App use	1.60*** (1.19–2.16)	1.62*** (1.20–2.19)	1.10* (1.01–1.19)	1.04 (0.95–1.14)
Mobile medical App use	1.12 (0.85–1.47)	1.12 (0.85–1.47)	1.11 (0.97–1.26)	1.16* (1.01–1.34)
Seeking behavior				
Online seeking for symptoms	4.99*** (2.26–10.98)	4.91*** (2.18–11.09)	2.50*** (1.74–3.60)	2.02*** (1.34–3.04)
Online seeking for services	6.86*** (2.41–19.52)	7.21*** (2.50–20.91)	2.42*** (1.65–3.54)	1.95** (1.28–2.99)
Perception				
Perceived trustworthiness of searching results	3.42*** (1.80–6.47)	2.86** (1.43–5.72)	2.00*** (1.37–2.93)	1.54 (0.99–2.39)

^a^Multivariate logistical analyses of STI testing controlled age, education, income, condom use and sex partner number.

^b^Multivariate logistical analyses of STI physician visiting controlled age, education, income, condom use, sex partner number, HIV status and syphilis status.

^c^aOR refers to adjusted odds ratio

*p <0.05, **p<0.01, ***p<0.001

## Discussion

This study examines YMSM’s online health information seeking behavior and its relationship with offline STI physician visits. This study extends the literature by exploring the role of social media as an emerging source of sexual health information. A majority of study participants had used online search engines and various forms of social media to seek sexual health information. YMSM considered gay mobile apps to be trustworthy sources of sexual health information, and utilized apps to seek health services. We also observed an association between online information seeking and offline physician visits, and found high rates of interest and willingness to use a MSM-friendly physician finding function.

We found that a majority of YMSM used generic social media and gay apps to seek sexual health information. This finding is consistent with prior surveys that have supported the emerging role of social media as searching tools [15, 23, 43, 44]. Compared with obtaining information from clinics and other institutions, online STI information seeking may be preferable because of its relatively anonymous, convenient, and confidential search process [45–47]. Social media platforms extend these advantages with increased adaptability, usability, and customizability [48]. Users can generate content and improve information quality through repeated, iterative processes [49, 50]. Individuals tend to have more trust in information shared by friends and acquaintances on social media compared with conventional sources [51]. With increasing integration of social media into daily communication habits [52], social media platforms represent novel opportunities for STI information dissemination.

YMSM indicated high levels of trustworthiness of STI information on gay mobile apps. Gay mobile apps were next only to authoritative governmental websites in the perceived trustworthiness. High levels of media use may build trust in certain media platforms [53, 54]. The most popular gay mobile app in China is accessed on average 227.5 times a week per user [55]. Gay mobile apps also expand opportunities for sexual health services to reach YMSM who may fear disclosing their same-sex behaviors in other contexts [56, 57]. MSM-exclusive platforms may also provide a feeling of safety among YMSM, and thus build trustworthiness toward the information on gay mobile apps [58]. Information about HIV/STIs and sexual health services on gay mobile apps can increase YMSM’s exposure and access to guidance and services of prevention, linkage to care, and treatment.

As YMSM have demonstrated high rates of Internet use for information seeking [5], improving access to offline healthcare services through online information is critical. In this study, we observed that online information seeking among YMSM was correlated with offline STI physician visits. In particular, seeking for symptoms was strongly associated with offline STI physicians, indicating that YMSM tend to more likely visit physicians in case of symptoms than screening of asymptomatic STI. Online information-based interventions have been implemented in many fields to promote health care [59, 60], including increasing HIV/STI testing [61] and antiretroviral therapy (ART) adherence [62]. Online information seeking may help empower YMSM with medical literacy, enable self-advocacy, and increase confidence before seeing a physician [63, 64]. Nonetheless, the prevalence of misinformation online can also make information seeking difficult [8]. Linking the high-quality, and accurate information to offline health services for YMSM will be critical in developing potential social media health interventions.

Most men were interested and willing to use a MSM-friendly physician finding function within gay-specific apps. This finding highlights the need for creating a friendly and comfortable environment to provide clinic services for MSM [65]. In the context of decentralization of health services, informing and suggesting STI physicians based on the geographical locations within gay-specific apps can improve healthcare accessibility and approachability [7]. In addition, many YMSM may not access health services in fear of being outed and healthcare discrimination [66]. Identifying and recommending MSM-friendly physicians can remove some barriers that defer YMSM to visit a physician and access healthcare resources.

This study has several limitations. First, participants were all recruited through social media platforms with internet access and social media use, which may overestimate the rate of online information seeking. Second, the participants were required to have visited a physician in the past 24 months; this may also result in an overestimate of the rate of visiting physicians. Third, participants were relatively well-educated, had higher income and had higher proportion living in Eastern China, and our findings may not be generalizable in other contexts or settings. Fourth, our survey did not distinguish those who sought STI information online once or repeatedly sought STI information online. Fifth, this study used a cross-sectional survey and thus could not determine any causal relationships. Finally, men self-reported sexual behaviors and healthcare seeking behaviors, introducing potential recall or social disability bias.

## Conclusion

Gay mobile apps and other social media platforms represent a unique opportunity to engage YMSM at high risk for HIV and STIs. Social media can be leveraged to disseminate STI information to YMSM. In addition, social media can be utilized to promote sexual health services, such as physician-finding functions. Our findings highlight the need to develop strategies to link YMSM to MSM-friendly physicians and health services.

## Abbreviations

aOR: Adjusted odds ratio
CHERRIES: Checklist for Reporting Results of Internet E-Surveys
HIV: Human immunodeficiency virus
MSM: Men who have sex with men
OR: Odds ratio
STI: Sexually transmitted infections
YMSM: Young men who have sex with men
